# Can Anthropomorphic Interfaces Improve the Ergonomics and Safety Performance of Human–Machine Collaboration in Multitasking Scenarios?—An Example of Human–Machine Co-Driving in High-Speed Trains

**DOI:** 10.3390/biomimetics10050307

**Published:** 2025-05-11

**Authors:** Yunan Jiang, Jinyi Zhi

**Affiliations:** Department of Industrial Design, Southwest Jiaotong University, Chengdu 610031, China

**Keywords:** human–computer collaboration, high-speed trains, anthropomorphic icons, driving tasks, cognitive performance

## Abstract

High-speed trains are some of the most important transportation vehicles requiring human–computer collaboration. This study investigated the effects of different types of icons on recognition performance and cognitive load during frequent observation and sudden takeover tasks in high-speed trains. The results of this study can be used to improve the efficiency of human–computer collaboration tasks and driving safety. In this study, 48 participants were selected for a simulated driving experiment on a high-speed train. The recognition reaction time, operation completion time, number of recognition errors, number of operation errors, SUS scale, and NASA-TLX questionnaire for the icons were all analyzed using analysis of variance (ANOVA) and the nonparametric Mann–Whitney U test. The results show that anthropomorphic icons can reduce the drivers’ visual fatigue and mental load in frequent observation tasks due to the anthropomorphic facial features attracting driver attention through simple lines and improving visual search efficiency. However, for the sudden takeover human–computer collaboration task, the facial features of the anthropomorphic icons were not recognized in a short period of time. Additionally, due to the positive emotions produced by the facial features, the drivers did not perceive the suddenness and danger of the sudden takeover human–computer collaboration task, resulting in the traditional icons being more capable of arousing the drivers’ alertness and helping them take over the task quickly. At the same time, neither type of icon triggered misrecognition or operation for sufficiently skilled drivers. These research results can provide guidance for the design of icons in human–computer collaborative interfaces for different types of driving tasks in high-speed trains, which can help improve the recognition speed, reaction speed, and safety of drivers.

## 1. Introduction

High-speed trains are an important form of public transportation [1], and they are often operated for long periods of time and across multiple regions. A high-speed train requires collaboration between the self-driving system and the driver during long driving hours and harsh environmental operation scenarios to reduce the driver’s cognitive load and task stress. Compared to other autonomous driving systems, such as self-driving cars [2], automated delivery robots [3], unmanned aerial vehicles [4], and self-driving boats [5], high-speed trains tend to be more difficult and complex to operate due to their length, speed, and duration of travel. As a result, most high-speed trains are now equipped with automatic train operator (ATO) systems that emphasize human–machine collaboration for co-driving rather than relying entirely on autopilot systems. Therefore, improving the performance of the automatic driving of high-speed trains requires improving the safety and usability of human–machine collaboration.

In the human–machine cooperative driving of trains, the most common task performed by the driver is observation. This includes observing the road conditions, observing the driving specifications ahead, and observing the train status information provided by the automated driving system through the Human–Machine Interface (HMI) screen. A driver’s vision needs to constantly switch between the road ahead and the HMI screen. Prolonged switching can quickly lead to visual fatigue, which affects the efficiency of human–machine collaboration and raises concerns related to driving safety [6]. In addition, railroad safety regulations require drivers to continuously press a pedal to indicate that they are awake and alert to avoid driver fatigue and distraction. However, this also means that drivers are subjected to significant biomechanical loads [7]. This visual fatigue and biomechanical load pose a challenge to the driver‘s cognitive efficiency and cognitive ability [8]. Train drivers must always maintain good cognitive abilities to stay focused and awake and ensure they are able to react quickly and correctly in unexpected situations when an automated driving system requires collaboration, such as when faced with train failures, network interruptions, or signaling anomalies [9]. Therefore, the driver must maintain a high level of cognitive ability during the collaborative human–machine train-driving process. There are two pathways that can be used to improve the cognitive ability of drivers. One pathway is to improve a train driver’s cognitive ability and attention while driving through professional and effective pre-driving learning and training, such as self-driving train-driving knowledge training, simulated driving, and attention training [10,11]. Another path involves improving driver recognition efficiency and reducing cognitive load during driving, such as through multi-modal interface information cues [12], more easily recognizable information, or clearer operating instructions to help train drivers for better cognitive performance. This study focuses on improving the effectiveness of human–machine collaboration by enhancing driver recognition. The information transfer of human–machine collaboration was mainly accomplished through the HMI, with the icons serving as the key factors carrying important information. Thus, it was reasoned that the recognition efficiency of the icons could be improved to effectively reduce the cognitive load of the driver and improve the safety and efficiency of human–machine collaboration in the self-driving train.

The design of icons plays a key role in influencing the efficiency of human recognition of information [13,14]. Icon design can facilitate the ability to quickly locate key information in the interface and draw attention to icons with appealing features [15]. Anthropomorphism refers to the tendency of humans to attribute human characteristics, intentions, and behaviors to non-human objects, such as products [16]. Based on this, adding human characteristics, intentions, or behaviors to an icon can anthropomorphize the icon, as demonstrated in Duan’s study [17]. In recent years, a series of studies have proven that anthropomorphic icons have advantages in recognition efficiency relative to traditional icons. For example, Guthrie et al. [18] found that anthropomorphic icons allowed users to better understand things that were previously difficult to understand by presenting them in symbolic terms that the users understood and were familiar with (Guthrie et al. [18]); this also made human–computer communication friendlier [19,20]. Moreover, Cao et al. [21] found that the addition of anthropomorphic elements to the design of weather app icons could enhance the interaction experience with users and improve their cognitive efficiency. Another study by Cao et al. [19] showed that anthropomorphic app icons were not only more attractive but also improved user cognitive attention. Anthropomorphic features can trigger positive attention and emotional experiences [21,22]. However, to the best of the authors’ knowledge, despite the fact that current high-speed trains are very advanced in terms of passenger interface and appearance, the icons used in human–computer collaboration driving systems are still traditional icons that have been in use for decades and as such, lack experience-based design and have poor usability [23]. Moreover, it is unclear how anthropomorphic icons affect human–computer collaboration in high-speed-train driving. To better determine the potential for anthropomorphic icons to enhance the safety and usability of high-speed-train driving, in this study, we investigated the impact of anthropomorphic icons on human–machine collaboration performance in the context of high-speed trains.

The tasks of high-speed-train autopilot human–machine collaboration can be categorized into two main categories. The first category is the daily observation of information such as autopilot status and road conditions. The second category consists of emergency takeover operations when the autopilot system malfunctions or emergency alarms go off [24]. Both task categories are important factors that affect the safety and efficiency of autonomous driving in high-speed trains. Most of the current research on reducing the cognitive load to improve cognitive performance in high-speed-train driving is insufficient, with most studies having been conducted on the takeover task during driving [25,26,27,28,29]. In contrast, there are only a small number of studies that address the first task, i.e., observation [30]. This means there is a lack of research on the first task category, and further research is needed to investigate the cognitive load and performance of the supervision and observation tasks during driving, which is another goal of this study.

**Figure 1:**
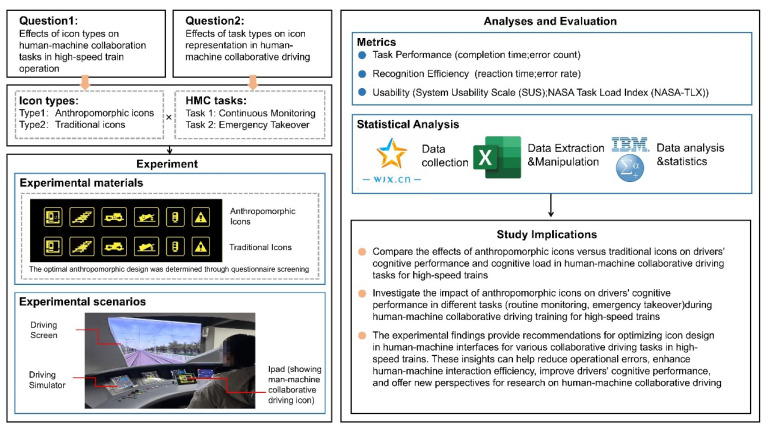
Research framework.

Given that current research on the performance of anthropomorphic icons during the human–machine collaborative automated driving of high-speed trains is incomplete, especially regarding the effects of cognitive performance and cognitive load on different driving tasks, this study makes the following major contributions (Figure 1 illustrates the main framework of this study):

(1) The effects of anthropomorphic and traditional icons on the cognitive performance and cognitive load of drivers during human–machine collaborative driving tasks in high-speed trains were compared;
(2) The effects of anthropomorphic icons on the cognitive performance of drivers in different tasks (i.e., daily observation and emergency takeover) during high-speed-train human–machine collaborative driving training were investigated;
(3) The results of the experimental study provide several suggestions for the optimal design of icons in human–machine interaction interfaces for various human–machine collaborative driving tasks in high-speed trains. These suggestions can be used to reduce the probability of errors during train operation, improve the efficiency of human–machine interaction, enhance the cognitive level of the drivers, and provide a new way of thinking about the study of human–machine collaborative driving.

## 2. Research Method

### 2.1. Participants

A total of 48 railroad school students were recruited as participants in this experiment, comprising 24 men and 24 women, and divided into four groups with the same number of men and women in each group. All subjects were between 18 and 30 years of age (M = 22.3; SD = 1.9), and all subjects had received at least 3 months of training in the appropriate driving tasks and had passed the school train-driving course. All participants were informed about the purpose of this experiment, and all subjects had normal or corrected vision and no known neurological disorders.

### 2.2. Experimental Equipment

**Figure 2:**
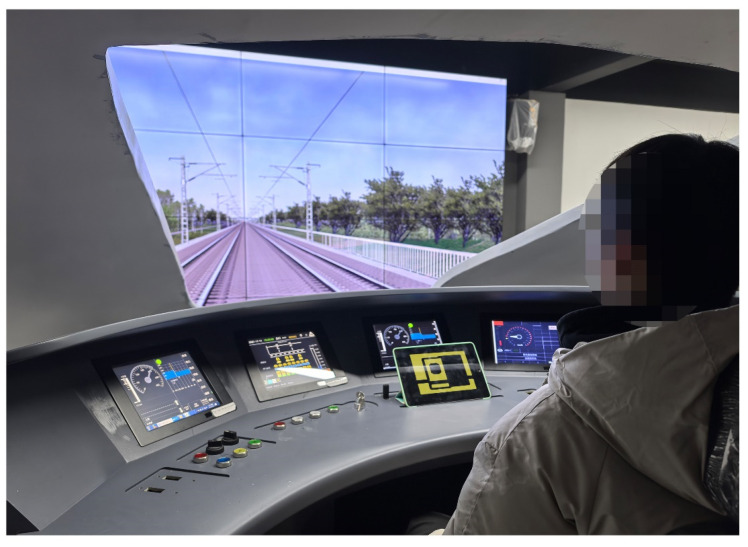
High-speed-train human–machine cooperation simulation driving platform.

**Figure 3:**
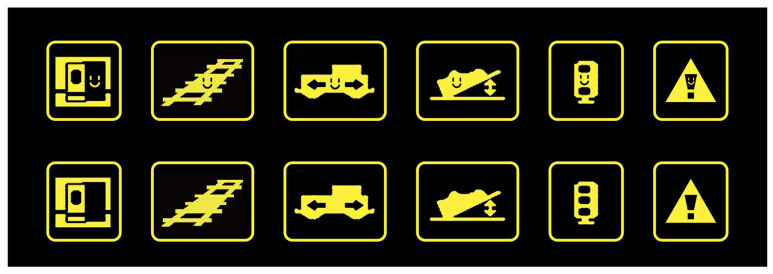
Icons for human–machine collaborative driving tasks. (Anthropomorphic icons are in the first row, and traditional icons are in the second row.)

The following equipment was employed in this study:

(1) A high-speed-train human–machine co-operation simulation driving platform. This platform was built utilizing a fan-shaped console, four ZhiXianDa QC1009 touch screens (13 inches), one iPad tablet, and a combination of 16 large screens. The buttons on the fan-shaped console included most of the functions used for driving high-speed trains, and participants needed to be based on the icon in the console to produce the corresponding response. The iPad tablet was used to simulate a high-speed-train human–machine interface display (Figure 2);
(2) High-speed-train human–machine collaboration icon material. Two different high-speed-train driving icon materials (anthropomorphic icons and traditional icons) were designed using Photoshop CC 2019 software, with the traditional icon being the icon used in the Chinese CRH380B high-speed train. The anthropomorphic icon was designed with reference to Duan’s research [17]. In this study, the anthropomorphism of the icon was scored using a seven-point Likert scale on the dimensions of “free will”, “consciousness”, “one’s own thoughts”, “emotion”, and “intention.” According to the scoring results, the smiling face graphics strongly increase anthropomorphism. Based on this, this study used the addition of smiley face graphics to perform icon anthropomorphic expressions. Each icon material set contained two different types of high-speed-train driving scenarios: a scenario requiring the frequent observation and identification of icons and a scenario requiring the emergence of unexpected situations and rapid action. The size and meaning of the two icons were the same (Figure 3). The icon material was rendered by importing it into the iPad;
(3) Other equipment: Two Sony cx900e camera DVs were used to record the entire experiment.

### 2.3. Variable Design

The independent variables were the different types of high-speed-train human–machine collaborative interface icons and driving scenarios. The types of icons were divided into anthropomorphic icons and traditional icons, and the human–machine collaborative driving tasks were divided into types of tasks: the first required the icons to be frequently observed and their meanings learned, while the second did not require frequent observation but did require quick and correct operation after the appearance of the icons, i.e., a sudden takeover task. The two sets of independent variables were combined to form four icon task groups, namely, an anthropomorphic icon-frequent observation group, an anthropomorphic icon-sudden takeover group, a traditional icon-frequent observation group, and a traditional icon-sudden takeover group.

The dependent variables included the reaction time, completion time, number of errors, and SUS usability scale. The completion time captured both the reaction time and operational efficiency across the icon types and driving task scenarios. The reaction time was the most direct indicator of recognition efficiency. The number of errors could effectively reflect the difficulty and efficiency of recognizing the meaning of icons, and the SUS usability scale reflected the subjective assessment of the usability of different types of icons in different driving scenarios. The specific indicators were as follows:

(1) Test completion time: This was taken as the time from when the icon requiring a quick response appeared to the time at which the subject completed the icon’s corresponding operation;
(2) Recognition reaction time: This was taken as the time from when the icon requiring frequent observation appeared to the time at which the subject selected the meaning of the corresponding icon;
(3) Number of operation errors: After the appearance of an icon that requires a quick response, the subject conducts the corresponding operation. The higher the number of operation errors, the lower the recognition efficiency of the icon and the more ambiguous the meaning;
(4) Number of recognition errors: After an icon that requires frequent attention appears, the subject needs to select the corresponding icon’s meaning. The higher the number of recognition errors, the poorer the recognizability and meaning of the icon;
(5) SUS Usability Scale: This consisted of 10 questions with five evaluation ranges for each question (completely disagree, disagree, average, agree, and strongly agree);
(6) NASA-TLX Questionnaire: The NASA Task Load Index is a set of processes and criteria developed by NASA’s Ames Research Center for the multidimensional evaluation of workload [31], and it includes subjective measurements in terms of mental demands, physical demands, time demands, effort, performance, and frustration. Each question was rated on a scale ranging from 0 (very low) to 100 (very high).

### 2.4. Experimental Procedure

The experiment was conducted in the following steps:

(1) First, participants were given an explanation of the purpose and task of the experiment. They were then introduced to the two types of icons and the operations that they correspond to, after which the participants were tested to ensure that they were sufficiently familiar with the meanings of the icons and their corresponding operations to be able to participate in the driving simulation effectively. In addition, a pre-experiment was utilized to ensure that the participants fully understood the flow of the experiment to follow;
(2) The participants were then divided into four groups corresponding to the four combinations of different icon types and driving scenarios. There were 12 people in each group, with an equal number of men and women. The corresponding personal information was recorded;
(3) A high-speed-train driving simulator was used to simulate driving scenarios by playing train-driving videos. Participants were seated in front of the high-speed-train driving simulator, and an iPad was placed in front of the participants. The corresponding icon was selected to present the video playback according to the group of participants;
(4) The participants who needed to frequently observe the scene needed to pay attention to the train-driving environment and the iPad icons at the same time. When an icon in a group of icons began to flash, the respondents needed to select the correct icon meaning from the five possible icon meaning options. After selecting the correct icon meaning, the train simulation continued until all icon meanings were selected. Meanwhile, the participants who needed to react quickly to the scenario had to observe the train-driving video most of the time. When an icon appeared on the iPad, the respondents needed to quickly perform the operation corresponding to the icon on the train simulation driving platform;
(5) After the simulation was completed, the researcher calculated the reaction and completion times and recorded the number of errors;
(6) The researcher arranged for the participants to fill out the questionnaire and then conducted a short post-experiment interview to reflect on and conclude the experiment.

## 3. Results

The experimentally obtained data on recognition reaction time, operation completion time, number of recognition errors, number of operation errors, and questionnaire score were subjected to Shapiro–Wilk and variance alignment tests, and it was found that the data on recognition reaction time and operation completion time conformed to a normal distribution and had a normal variance alignment (*p* > 0.05); however, the data on the number of recognition errors, number of operation errors, and questionnaire score did not conform to a normal distribution or variance alignment (*p* < 0.05), and the number of errors took the form of count-type discrete data. Therefore, one-way analysis of variance (ANOVA) was used to analyze the recognition reaction time and operation completion time, whereas a nonparametric Mann–Whitney U test was performed on the experimentally obtained number of recognition errors, number of operation errors, SUS scale, and NASA-TLX scores.

### 3.1. Recognition Reaction Time

**Figure 4:**
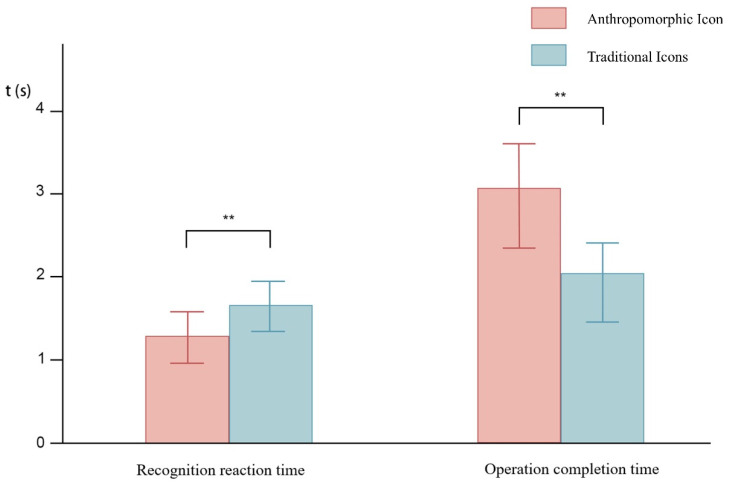
Average recognition reaction time and operation completion time for different icons in different driving tasks. (** represents *p* ≤ 0.01).

The one-way ANOVA analyzing the recognition reaction times of icons for frequent observation tasks found that there was a significant difference in the recognition reaction time for different icon types (*p* < 0.05), as shown in Table 1. A later comparison of the data reveals that the recognition reaction time for anthropomorphic icons for the frequent observation tasks was significantly smaller than that of traditional icons (*p* < 0.05). A comparison of the mean recognition reaction times of different icon types for the frequent observation task is shown in Figure 4.

### 3.2. Operation Completion Time

One-way ANOVA of the operation completion time after icon prompts for the sudden takeover task found that there was a significant difference in the operation completion time between different icon types (*p* < 0.05), as shown in Table 1. A later comparison of the data found that anthropomorphic icons had a significantly greater operation completion time than traditional icons (*p* < 0.05). A comparison of the average operation completion times of different icon types for the sudden takeover task is shown in Figure 4.

### 3.3. Number of Recognition Errors and Number of Operation Errors

Mann–Whitney U tests of the number of recognition errors in the frequent observation task and the number of operation errors after icon prompts for the sudden takeover tasks show that the number of both types of errors did not differ significantly (*p* > 0.05) for different icon types.

### 3.4. SUS Usability

**Figure 5:**
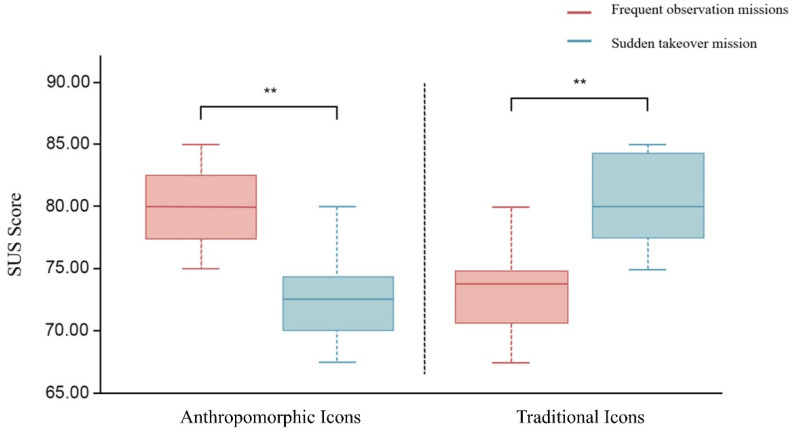
SUS scores for different types of icons for different driving tasks. (** represents *p* ≤ 0.01).

After the experiment was completed, the subjects were asked to fill out an SUS Usability Scale questionnaire corresponding to the group and task to measure their perceptions of icon usability. After transforming the SUS scores of 48 subjects, it was found using the Mann–Whitney U-test that there was a significant difference (*p* < 0.05) between the median SUS scores of the different icon types in different high-speed-train driving tasks, as shown in Table 2. For the anthropomorphic icons, the SUS scores in the frequent observation task were significantly higher than the SUS scores in the sudden takeover task (*p* < 0.05). However, for conventional icons, the SUS scores for the frequent observation task were significantly lower than the SUS scores for the sudden takeover task (*p* < 0.05). The median SUS scores for different icon types and driving tasks are compared in Figure 5.

### 3.5. NASA-TLX Scores

**Figure 6:**
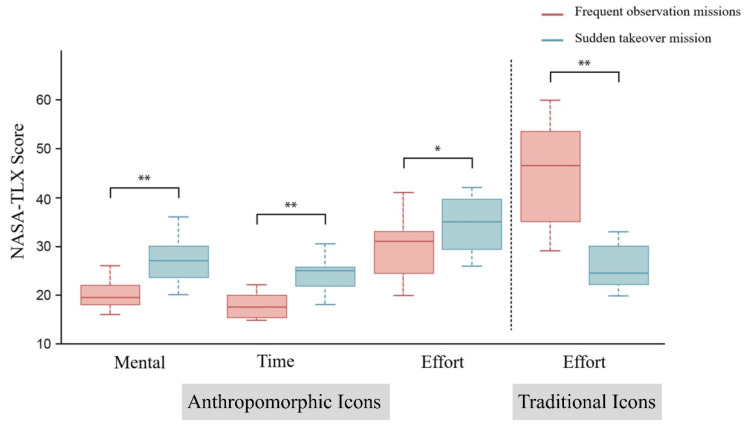
NASA-TLX scores for different types of icons for different driving tasks. (* represents *p* ≤ 0.05, ** represents *p* ≤ 0.01).

Upon completion of the experiment, the participants were administered the NASA-TLX questionnaire, which asked the subjects to use a scale of 0–100 to rate their subjective feelings regarding the mental demands, physical demands, time demands, effort, performance, and frustration related to the different iconographic interfaces and different high-speed-train driving tasks. A Mann–Whitney U-test of the NASA-TLX questionnaire scores collected from the 48 subjects found that there were significant differences (*p* < 0.05) in mental demand, time demand, and effort for different high-speed-train driving tasks, as shown in Table 2. A comparison between the two driving tasks reveals that anthropomorphic icons had significantly lower median brain demand, time demand, and effort scores in the frequent observation task than in the sudden takeover task (*p* < 0.05). At the same time, the traditional icons significantly differed (*p* < 0.05) in terms of effort in different driving tasks. By comparing the median effort scores for the two driving tasks, it was found that traditional icons required significantly more effort in the frequent observation task than in the sudden takeover task (*p* < 0.05). A comparison of the median NASA-TLX scores for different icon types and high-speed-train driving tasks is shown in Figure 6.

## 4. Discussion

This section presents important findings obtained from the analysis of experimental data from high-speed-train human–machine collaborative driving tasks. It explores the effects of different types of icons on driver cognitive performance and load during different types of driving tasks in human–computer collaborative driving in high-speed trains. Questions from some of the users after the experiment and feedback about how they felt about the experiment helped explain some of the potential reasons for the experimental results.

A comparison of the recognition completion time for the frequent observation task and the operation completion time for the sudden takeover task with different types of icons reveals that, in the frequent observation task, icon recognition occurred more rapidly when using anthropomorphic icons than when using traditional icons. This might have been because the facial features of the anthropomorphic icons were able to attract the driver’s attention through simple lines. Anthropomorphic icons were also found to have a greater ability to capture attention in a study by Cao [19]. It has also been demonstrated that anthropomorphic features can stimulate positive emotional experiences [21,22], as well as that emotional information can be processed quickly and enhance visual perception [32]. This also explains the fact that in the case of anthropomorphic icons, drivers could quickly recognize the icons, obtain the relevant information, and undertake the corresponding operation. Moreover, the frequent observation task requires multiple instances of recognition and rapid understanding of the current icon meanings to obtain a responsive train-driving state. Anthropomorphic icons may produce positive emotions in addition to faster recognition, which may help a driver obtain a better working state [33]. Therefore, anthropomorphic icons are most suitable for frequent observation human–machine collaboration tasks.

In the case of unexpected human–machine collaboration tasks that require a takeover, traditional icons can help a driver complete the takeover operation required by an unexpected cue more quickly than anthropomorphic icons. This is consistent with Shang et al.’s study [34], which found that cues with rapid stimuli conveyed through the facial information of anthropomorphic icons might not be effective in getting the attention of drivers. For a sudden takeover task, the takeover icon is presented quickly and suddenly when there is an emergency, and the driver may not be able to quickly process the facial information featured on an anthropomorphic icon. However, after the icon has been present for a longer period of time, the driver will notice the facial features of the anthropomorphic icon. These types of features usually produce positive, happy emotional feedback [22], but this may lead to a decrease in the driver’s alertness [35], which may, in turn, prevent the driver from responding quickly to the takeover task. This was also confirmed in the subjects’ post-experimental interviews. In these interviews, subjects in the anthropomorphic icon group who performed the sudden takeover human–machine collaboration task generally mentioned that they were unable to quickly perceive that the current driving state was dangerous after seeing the icon and thus lacked a sense of urgency when performing the takeover operation. This then led to a relatively slow takeover operation. Thus, for a frequent human–computer collaborative task requiring fast recognition, attention could be captured by the facial information features of anthropomorphic icons. For icon cues in human–machine collaborative tasks that require a rapid response and immediate attention, traditional icons can better convey the dangerous and urgent status of the train.

A comparison of the number of recognition errors and the number of takeover operation errors for different types of icons was carried out for two human–machine collaborative tasks. There was no significant difference in the recognition errors and takeover operation errors between anthropomorphic icons and traditional icons. This might have been due to the fact that the subjects were well instructed and trained to perform accurate recognition, as well as the corresponding operation, regardless of the icons. This was also consistent with the real human–machine collaborative driving task, about which the subjects already possessed better theoretical knowledge when driving. This was verified by McDougall et al. [36]. In their research, the advantage of reducing complexity for recognition diminished as familiarity with the icons increased. This is also consistent with the study by Shen et al. [37], in which greater familiarity with the icons led to better recognition, but as the familiarity increased, it helped less and less. This was confirmed in our post-experiment interviews with the subjects. The subjects in both groups told us that once they had responded to the meanings expressed by the icons, they were able to explicitly understand the corresponding actions and behaviors. This finding suggests that for tasks such as human–machine collaborative driving in high-speed trains, which require extensive training and assessment, when the driver’s proficiency reaches a certain level, more attention needs to be paid to a driver’s cognitive load and reaction speed when designing interfaces and icons, and determining how to capture the driver’s visual attention more quickly becomes increasingly important.

Comparisons of the SUS usability scale scores and NASA-TLX questionnaire scores were carried out for the two types of human–computer collaboration tasks and two types of icons. In terms of SUS usability, anthropomorphic icons had significantly higher usability scores than traditional icons in the frequent observation task, while traditional icons had significantly higher usability scores than anthropomorphic icons in the sudden takeover task. This was also consistent with the trend of the data related to recognition response and at the time of operation completion, and it could serve as a basis for some interpretation of each other. Additionally, for anthropomorphic icons, the brain demand, time demand, and effort results from the NASA-TLX questionnaire, as well as the brain demand results from the NASA-TLX questionnaire for traditional icons, could be further explained. For tasks that require frequent observation, drivers need to constantly perform visual searches, and their visual attention has to switch frequently between the interface and the surrounding environment. According to Duan’s research [17], anthropomorphic icons have a greater effect on spatial attention than non-anthropomorphic icons and are, therefore, more suitable for human–computer collaborative tasks, such as frequent observation tasks with multiple information displays, and these icons can thus reduce visual fatigue more than traditional icons. Moreover, elevated visual fatigue imposes a greater cerebral load [38]. This also explains why, for frequent observation tasks, subjects in a traditional icon group will experience higher brain power and time demands and consequently exert greater effort. In contrast, for sudden takeover tasks, being able to quickly make the driver aware of the current dangerous situation and have them complete the takeover is of paramount importance. The positive emotions that may be associated with anthropomorphic icons are not suitable for sudden takeover tasks in train driving, which provide a short-term stimulus and require a rapid response. The emotional effect of anthropomorphic icons in short-term stimulus tasks can even lead to a higher cognitive load due to the need for sufficient time to process the information [39,40]. Based on this, for tasks that require frequent observation, the visual fatigue and cerebral load of drivers can be reduced by anthropomorphized icon design, but for sudden takeover tasks that require drivers to respond quickly, traditional icons should be used to ensure rapid cueing and reactions.

## 5. Conclusions

In this study, we compared and determined the differences in recognition efficiency, recognition errors, and icon availability of different icon types for drivers in two typical train-driving human–machine collaboration tasks, namely, frequent observation tasks and sudden takeover tasks, through icon recognition experiments. The results show that for frequent observation tasks, anthropomorphic icons have a significant advantage over traditional icons in terms of recognition reaction time, SUS availability, brain demand, time demand, and effort. The anthropomorphic facial features in anthropomorphic icons can attract driver attention through simple lines and improve visual search efficiency, which can reduce driver visual fatigue and cerebral load. However, it is difficult to quickly recognize the facial features of anthropomorphic icons, which is problematic for sudden takeover tasks. Moreover, in these tasks, due to the positive emotions produced by the icon’s facial features, the driver may not perceive the suddenness and danger of the sudden takeover task; as a result, the traditional icons are more capable of arousing the driver’s alertness and encouraging them to take over the task quickly. In the case of high-speed-train driving, which requires a high level of proficiency, there are no significant differences in the effect of icons on recognition performance. Therefore, more attention should be paid to a driver’s cognitive performance and response time in the design of icons. The results of this study can provide guidance for high-speed-train human–machine collaboration tasks when designing appropriate icon types for different driving tasks. The design and display of icons should be customized according to the type of task to ensure that drivers can access key information in the shortest amount of time and optimize the cognitive performance and response time of drivers, thus improving the efficiency and safety of human–machine collaboration in driving high-speed trains.

There are several limitations to this study. First, in this study, the effects of icon color were not investigated, with the color of the anthropomorphic icons corresponding to the current traditional icons. Second, the number of icons should be further increased to explore the effects of anthropomorphic icons on more operations in high-speed-train driving. Also, there is a lack of variability in the subjects’ driving experience, and drivers with different driving experiences may have different research findings. Therefore, in future research, we plan to increase the variety of icon colors and the number of icons. We also plan to further refine the specific tasks in the two driving tasks to provide better theoretical support for the application of anthropomorphic icons in high-speed-train driving tasks.

## Figures and Tables

**Table 1:** Means, standard deviations, and one-way analysis of variance for recognition reaction times in frequent observation tasks and completion times in sudden takeover tasks across icon types.

	Icon Type	Mean ± SD	F	*p*
Recognition reaction time for frequent observation tasks	Traditional Icons	1.65 ± 0.30	8.968	0.007 **
	Anthropomorphic Icons	1.28 ± 0.31		
Operational completion time for sudden takeover tasks	Traditional Icons	2.04 ± 0.37	31.999	0.000 **
	Anthropomorphic Icons	3.07 ± 0.51		

** represents *p* ≤ 0.01.

**Table 2:** Median and descriptive statistics of same-session SUS and NASA-TLX scores for frequent observation tasks versus sudden takeover tasks across different icon types.

	Type of Task	Median	Z	*p*
Anthropomorphic Icons SUS Score	Frequent observation missions	80.000	−3.799	0.000 **
	Sudden takeover mission	72.500		
Traditional Icons SUS Score	Frequent observation missions	73.750	−3.356	0.001 **
	Sudden takeover mission	80.000		
Anthropomorphic Icons NASA-TLX Brain Score	Frequent observation missions	19.500	−3.454	0.001 **
	Sudden takeover mission	27.000		
Anthropomorphic Icons NASA-TLX Time Score	Frequent observation missions	17.500	−3.775	0.000 **
	Sudden takeover mission	25.000		
Anthropomorphic Icons NASA-TLX Effort Score	Frequent observation missions	31.000	−2.115	0.034 *
	Sudden takeover mission	35.000		
Traditional Icons NASA-TLX Effort Score	Frequent observation missions	46.500	−3.907	0.000 **
	Sudden takeover mission	25.500		

* represents *p* ≤ 0.05, ** represents *p* ≤ 0.01.

## Data Availability

The data presented in this study are available upon request from the corresponding author. The data are not publicly available due to privacy or ethical restrictions.
